# Inflammatory choroidal neovascularization associated with immunoglobulin G4-related disease: a case report

**DOI:** 10.1186/s13256-020-02431-8

**Published:** 2020-07-05

**Authors:** Chirag Jhaveri, Giovanni Campagna

**Affiliations:** 1 Retina Consultants of Austin and Retina Research Center, 3705 Medical Parkway, Suite 410, Austin, TX 78705 USA; 2 Department of Ophthalmology, The University of Texas at Austin Dell Medical School, Austin, TX USA; 3 Transitional Year Residency Program, The University of Texas at Austin Dell Medical School, Austin, TX USA

**Keywords:** Choroidal neovascular membrane, IgG4, Immunoglobulin G4-related disease, Immunoglobulin G4-related ophthalmic disease, Inflammatory choroidal neovascularization

## Abstract

**Background:**

Immunoglobulin G4-related disease is a recently recognized condition with pathologic features that are consistent across a wide range of organ systems. Orbital manifestations of this disease entity typically involve the lacrimal gland and lacrimal duct, extraocular muscles, orbital soft tissue, and sclera. Here, the authors report the first known case of inflammatory choroidal neovascular membrane associated with immunoglobulin G4-related disease and offer suggestions for clinical management of this enigmatic condition.

**Case presentation:**

A 38-year-old Caucasian man with a history of recurrent tonsillitis presented with blurry vision in his left eye of 1-week duration and was diagnosed as having inflammatory choroidal neovascular membrane. An infectious workup was negative, but his serum immunoglobulin G4 level was elevated at 248 mg/dL (reference 4–86), and a subsequent tonsillectomy for a repeat episode of tonsillitis revealed increased immunoglobulin G4 staining on histopathology, thus confirming the diagnosis of immunoglobulin G4-related disease. The inflammatory choroidal neovascular membrane was treated with intravitreal bevacizumab injections and orally administered prednisone resulting in improved visual acuity and choroidal neovascular membrane regression. He later received rituximab infusions for immunoglobulin G4-related disease.

**Conclusions:**

We report a case of choroidal neovascularization associated with immunoglobulin G4-related disease, a chronic inflammatory condition whose ophthalmic manifestations typically include dacryoadenitis, orbital myositis, or scleritis. This is the first reported instance of inflammatory choroidal neovascular membrane associated with immunoglobulin G4-related disease. Early detection of this disease is important to avoid organ damage and potential complications, so clinicians should maintain an index of suspicion for this condition when inflammatory choroidal neovascular membrane is observed.

## Background

A choroidal neovascular membrane (CNVM) is a pathological growth of new blood vessels that originate in the choroid through a break in Bruch’s membrane into the sub-retinal pigment epithelium (RPE) space or sub-retinal space. The differential diagnosis of new CNVM is broad and includes a wide range of conditions such as age-related macular degeneration, myopic degeneration, and ocular inflammation [1]. When CNVM is associated with inflammatory conditions of the posterior segment, it is referred to as inflammatory CNVM. Inflammatory CNVM can occur from a disruption in the interface between Bruch’s membrane and the RPE, from an angiogenic stimulus mediated by local inflammation, or from a combination of both [2]. Classically, inflammatory CNVM presents as new-onset distortion or metamorphopsia, sometimes leading to diminution of vision or a scotoma. Due to a lack of randomized controlled clinical trials regarding treatment of inflammatory CNVM, there is no consensus or set of evidence-based guidelines for the management of this condition. Management approaches include observation, intravitreal anti-vascular endothelial growth factor (VEGF) injections, photodynamic therapy, corticosteroids and other immunosuppressive agents, and laser photocoagulation [1].

The authors report a case of a young adult man who developed inflammatory CNVM in his left eye (OS) associated with immunoglobulin G4 (IgG4)-related disease (IgG4-RD), a chronic immune-mediated fibroinflammatory systemic disease that can affect any organ in the body and is characterized by focal or diffuse organ infiltration by IgG4-bearing plasma cells. The inflammatory CNVM was treated with intravitreal bevacizumab injections and orally administered prednisone, resulting in improved visual acuity and ultimately CNVM regression. He later received rituximab infusions for IgG4-RD. This represents the first reported case of CNVM associated with IgG4-RD in the English ophthalmic literature and sheds light on an otherwise uncommon etiology of inflammatory CNVM. Intraocular findings in cases of IgG4-RD are variable and have been sparsely described until recently, and this case augments the growing body of literature by expanding the spectrum of disease presentation. This case should encourage clinicians to maintain an index of suspicion for IgG4-RD when inflammatory CNVM is observed.

## Case presentation

A 38-year-old Caucasian man presented with worsening central vision OS of 1-week duration. His medical history was significant for recurrent tonsillitis but otherwise negative. He had been diagnosed as having “strep throat” twice in the last 3 months and prescribed amoxicillin 500 mg twice per day for 10 days both times without formal rapid strep testing. He recovered from each instance of tonsillitis within 5–7 days. He had no history of ophthalmic conditions or prior surgery. He denied taking any daily medications or using eye drops. He grew up in Kansas City, Missouri, United States of America, and owned a dog but denied recent travel or exposure to ticks. He and his wife have lived in Austin, Texas, USA, for several years. He works as an information technology specialist. He is a former cigarette smoker who quit 8 years ago, he drinks alcohol socially, and he denied any history of illicit drug use. His family history is significant for maternal diabetes and paternal hypertension.

**Fig. 1:**
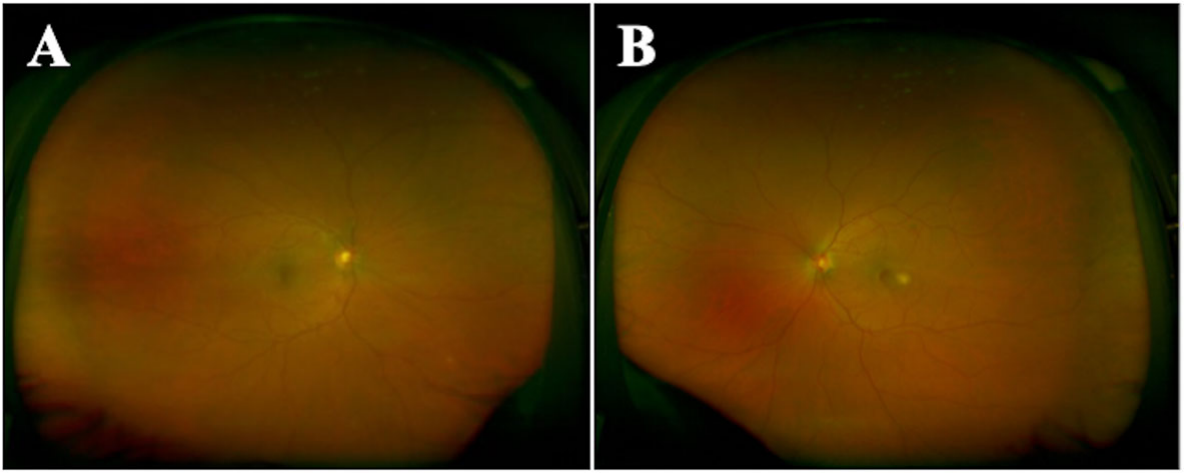
Fundus photography of the right eye (**a**) and left eye (**b**) with an ultra-widefield retinal camera. A small cream-colored sub-retinal lesion temporal to the fovea with a raised appearance is appreciated in the left eye

His uncorrected visual acuity was 20/20 in his right eye (OD) and 20/50 OS. His pupils were equally round and reactive, and intraocular pressures were normal. The ocular adnexa were normal on external examination. The anterior chamber and vitreous were quiet bilaterally. A dilated fundus examination OD was unremarkable. A dilated fundus examination OS showed a normal optic nerve, scant hyperpigmentation at the inferotemporal border of the disc, and a whitish sub-retinal lesion temporal to the fovea with a small associated hemorrhage. Ultra-widefield fundus photography is shown in Fig. 1. A general medical examination was unremarkable. He was afebrile with a temperature of 36.7 °C (98.0 °F), heart rate of 73 beats per minute, right arm cuff blood pressure of 113/70 mmHg, respiratory rate of 16 breaths per minute, and 98% oxygen saturation on room air. His oropharynx was clear without exudates, his lungs were clear to auscultation, his heart was without murmur or arrhythmia, he had no rashes, and all cranial nerves II–XII were intact with no gross motor or sensory deficits, and he had normal gait.

**Fig. 2:**
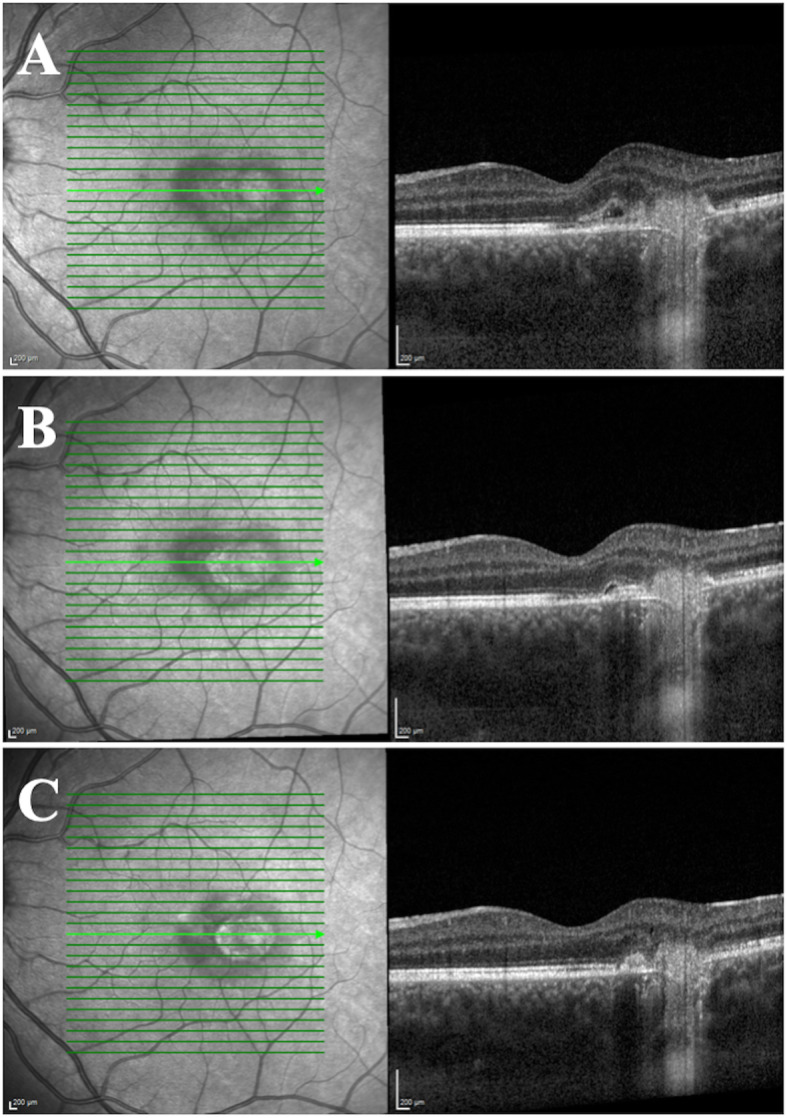
Optical coherence tomography of the macula of the left eye at initial presentation (**a**) showing a hyperreflective sub-retinal elevation with an associated small pocket of fluid and normal foveal contour. At the first follow-up visit 1 week after receiving an intravitreal anti-vascular endothelial growth factor injection, the lesion was largely resolved (**b**). Six months later, the choroidal neovascular membrane is reduced in size and there was no intra-retinal fluid (**c**)

**Fig. 3:**
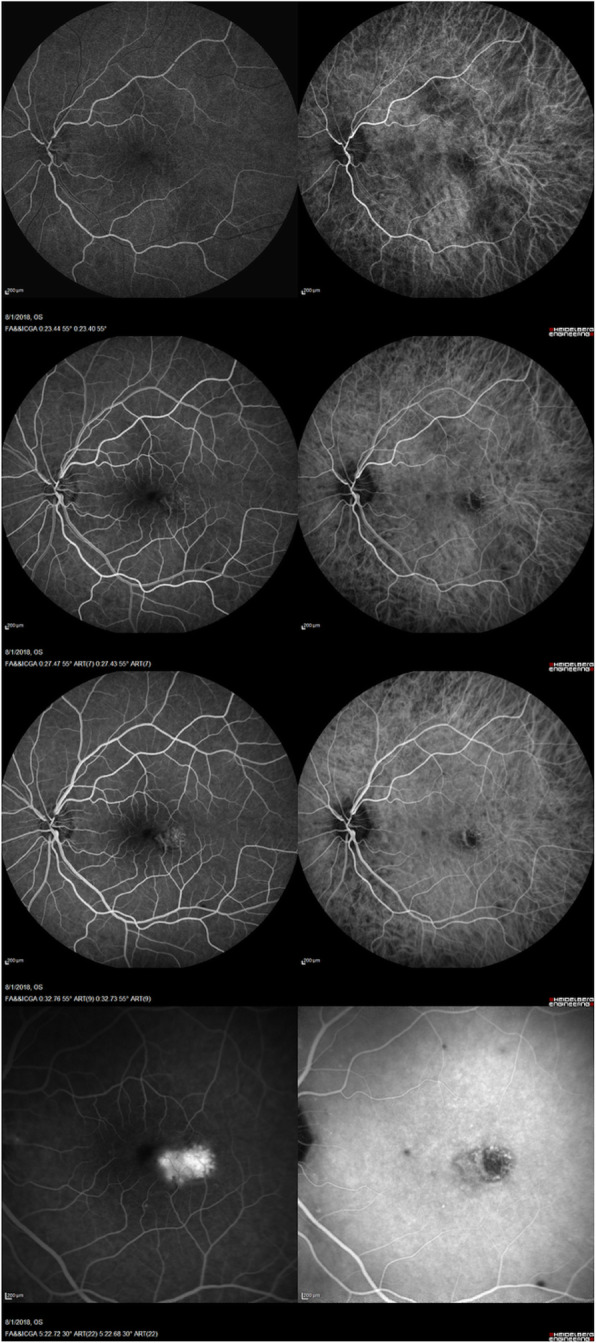
Fluorescein angiography (*left column*) and indocyanine green angiography (*right column*) of the patient’s left eye. Fluorescein angiography shows normal arteriovenous transit and a focal area of hyperfluorescence temporal to the fovea particularly in late-phase, consistent with a choroidal neovascular membrane, and transmission defects consistent with retinal pigment epithelium changes. Indocyanine green angiography shows early blockage with mild late leakage corresponding to an inflammatory lesion with a choroidal neovascular membrane as well as some isolated focal hypocyanescent areas

**Fig. 4:**
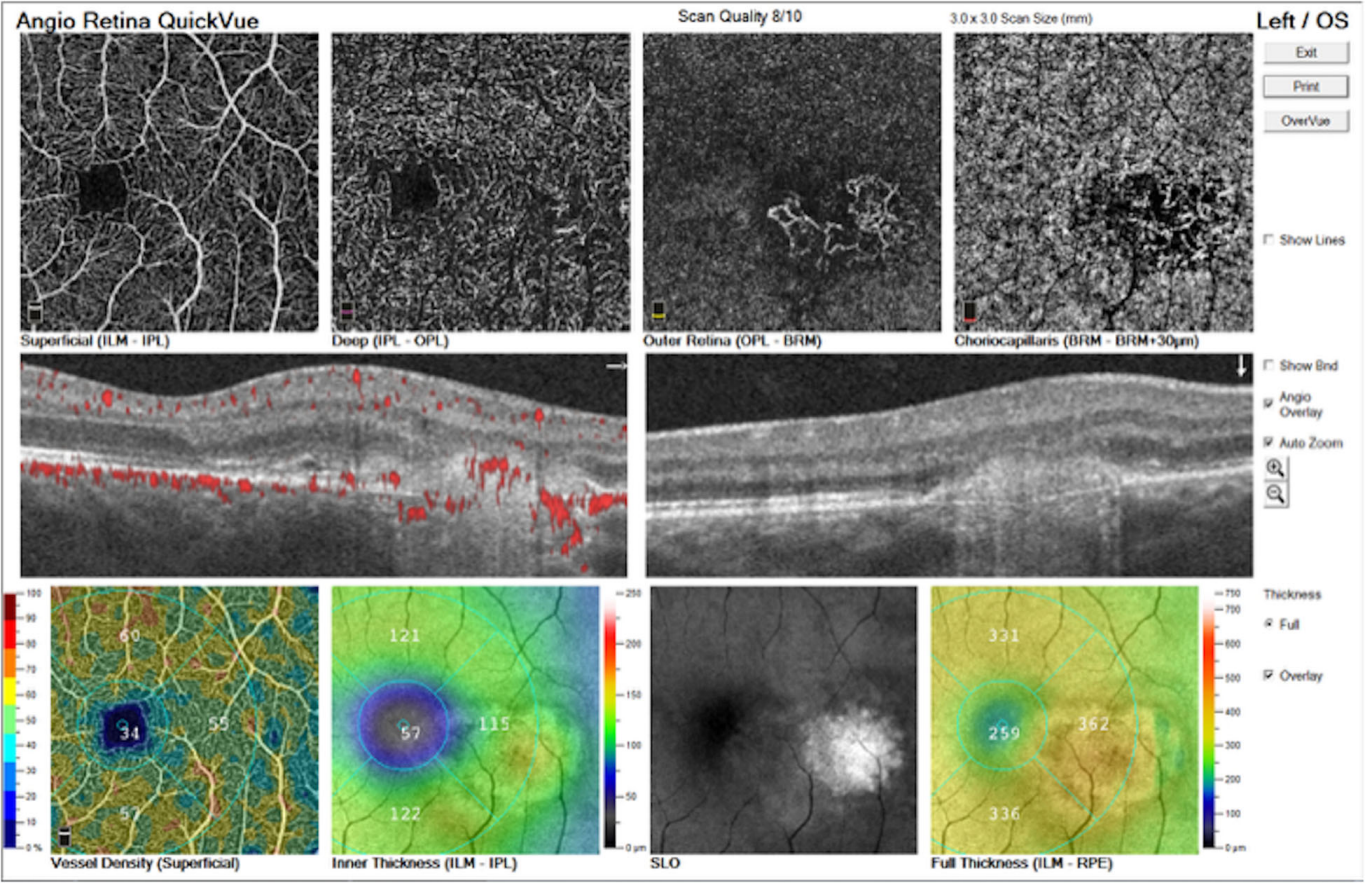
Optical coherence tomography angiography of the patient’s left eye. This shows a coralliform vascular complex in the outer retina that originated from within the choroid and traversed the retinal pigment epithelium and Bruch’s membrane into the sub-retinal space

Optical coherence tomography (OCT) (Fig. 2a) showed a hyperreflective sub-retinal elevation with an associated small pocket of fluid. Fluorescein angiography (Fig. 3) showed a focal area of hyperfluorescence temporal to the fovea in late phase. Indocyanine green (ICG) angiography (Fig. 3) showed early blockage with mild late leakage and isolated focal hypocyanescent areas. OCT angiography (Fig. 4) showed a coralliform vascular complex in the outer retina that originated from within the choroid and traversed the RPE and Bruch’s membrane into the sub-retinal space. His examination and ocular imaging were consistent with inflammatory CNVM OS with associated fluid collection, so he was initially treated with an intravitreal bevacizumab 1.25 mg/0.05 mL injection OS. Regarding his laboratory findings, his white blood cell count was 8.4 × 10^3^ per mm^3^, hemoglobin 12.5 g/dL, platelets 222 × 10^3^ per mm^3^, prothrombin time 14.5 seconds, international normalized ratio 1.26, blood urea nitrogen 9 mg/dL, creatinine 0.8 mg/dL, aspartate aminotransferase 22 u/L, alanine aminotransferase 27 u/L, and total bilirubin 0.5 mg/dL. Rapid plasma reagin, QuantiFERON-TB Gold, antinuclear antibody profile, and toxoplasmosis titers were non-reactive or unremarkable. His chest X-ray showed a calcified nodule in the upper lung field of his right lung, later confirmed with chest computed tomography.

One week after initial presentation and treatment with bevacizumab, his visual acuity improved to 20/25 OS, and OCT showed improvement of the sub-retinal fluid (Fig. 2b). A 2-week taper of orally administered prednisone starting at 60 mg per day and ending at 5 mg per day was prescribed to treat the inflammatory component of the CNVM. He was referred to pulmonology, and an additional workup revealed elevated serum IgG4 levels at 248 mg/dL (reference 4–86). He was also referred to otolaryngology for his history of recurrent tonsillitis, for which he underwent bilateral tonsillectomy. The specimen showed significant staining for IgG4 on histopathology. After completion of the first orally administered prednisone course, he developed left upper eyelid blepharitis, which did not resolve after initial treatment with antibiotic ointment and warm compresses. During the next 4 months, he received two more intravitreal bevacizumab 1.25 mg/0.05 mL injections for recurrence of sub-retinal fluid associated with inflammatory CNVM in the setting of biopsy-proven IgG4-RD, as well as a 1-month taper of orally administered prednisone starting at 60 mg per day and ending at 5 mg per day, after which the blepharitis resolved. He experienced improvement in his visual acuity with each injection. Six months after initial presentation, he was started on rituximab infusions 1000 mg every 2 weeks in order to decrease circulating IgG4 levels. The CNVM had decreased in size by this point (Fig. 2c). He is seen by his retina subspecialist (CJ) every 2 months for examination and anti-VEGF therapy as needed.

## Discussion and conclusions

In summary, we present the first reported case of inflammatory CNVM in the setting of IgG4-RD. Treatment was achieved with anti-VEGF injections as well as steroid tapers in order to stop sub-retinal fluid accumulation as well as inflammatory components, respectively. In this discussion we provide a thorough review of the natural history of IgG4-RD and describe how this case differs from what has been published in the literature regarding ophthalmic manifestations of the disease.

IgG4-RD is a recently recognized condition with pathologic features that are consistent across a wide range of organ systems. While the exact mechanism of the disease remains unknown, the prevailing contention is that IgG4-RD is mediated primarily by T helper type 2 cells, which secrete different interleukins that activate plasma cells to produce IgG4, as well as regulatory T cells [3]. The first reported instance of this condition was described by Sarles *et al.* [4] and involved a case of pancreatitis with hypergammaglobulinemia, but IgG4-RD has since been discovered as the cause of inflammatory disease in many organs including the central nervous system, orbits, ears, nose, throat, salivary glands, thyroid gland, lungs, kidneys, and others. Ophthalmic and orbital manifestations of IgG4-RD, now referred to as IgG4-related ophthalmic disease (IgG4-ROD), typically involve the lacrimal gland and lacrimal duct, extraocular muscles, orbital soft tissue, and sclera, as well as the cranial nerves and their branches [5, 6]. Whereas IgG4-RD typically occurs more often in men and targets middle-aged men [7], men and women tend to be affected equally in cases of IgG4-ROD [5]. Furthermore, in a recent single-center retrospective review, 71% of patients with biopsy-confirmed IgG4-ROD had bilateral ophthalmic or orbital disease, and 71% had extra-orbital involvement [8].

IgG4-ROD remains a diagnostic challenge for several reasons: (1) the epidemiology of the condition is poorly described due to its recent arrival in the diagnostic lexicon, (2) the presentation is variable, and (3) a large number of other inflammatory conditions are known to affect the orbit and adnexa. Ophthalmic manifestations of pathologically high levels of IgG4 include dacryoadenitis, blepharitis, scleritis, cranial nerve involvement, and orbital soft tissue inflammation [3, 5, 6, 8, 9]. Other documented presentations of IgG4-ROD include idiopathic orbital inflammation (IOI) and idiopathic orbital myositis [10]. Proptosis is a common feature in these two conditions. IOI can affect either the entire orbit or specific components such as the extraocular muscles, lacrimal system, optic nerve, or sclera. Histopathology demonstrates a benign, nonspecific, polymorphic inflammatory infiltrate, sometimes accompanied by sclerosis [10]. Without specific IgG4 staining, it is impossible to differentiate IOI from IgG4-RD, and it is possible that a substantial proportion of cases of presumed IOI are, in fact, IgG4-ROD. Idiopathic orbital myositis is known to commonly present with diplopia and pain with eye movements. In their review of 15 individuals with inflammatory CNVM, D’Souza *et al.* concluded that the presence of concurrent active inflammation is not an essential factor for development of inflammatory CNVM [11], and that CNVM by definition is merely a CNVM associated with a condition that may manifest as posterior segment inflammation, which is possible in IgG4-ROD. In the first published case of choroidal lesions associated with IgG4-ROD, no signs of intraocular inflammation were observed [12]. Intraocular inflammation in the form of anterior chamber cell and flare or vitritis was not observed in this patient, but these findings may be subtle, and our patient presented relatively late in the course of his symptomatology. Considering our patient’s history, ophthalmic examination, elevated serum IgG4 and confirmatory biopsy, and resolution of symptoms with intravitreal anti-VEGF injections and orally administered steroids, the authors are confident that IgG4-RD is the cause of this patient’s inflammatory CNVM and blepharitis OS. Therefore, this case report showcases an unusual presentation of IgG4-ROD and expands the current spectrum of this enigmatic condition.

Infectious etiologies of CNVM, such as syphilis, tuberculosis, and toxoplasmosis, were ruled out with a thorough laboratory workup. Although our patient was originally from the Midwestern USA, his ophthalmic examination was not consistent with presumed ocular histoplasmosis syndrome, which often presents with the clinical triad of: (1) discrete atrophic choroidal scars in the macula or periphery, (2) peripapillary atrophy, and (3) choroidal neovascularization. Our patient’s examination was devoid of atrophic choroidal scars, or “histo spots.” In addition, ocular histoplasmosis syndrome tends to produce early hypercyanescence on ICG angiography, but this patient’s ICG angiography revealed early blockage with late leakage and focal hypocyanescence. While the calcified pulmonary nodule was not biopsied, serum IgG4 levels and increased IgG4 staining on subsequent tonsillectomy histopathology confirmed the diagnosis of IgG4-RD in this patient. However, the presence of a pulmonary lesion does warrant consideration for histoplasmosis as well as sarcoidosis, which tend to have skin involvement (for example erythema nodosum) and bilateral pulmonary hilar adenopathy, and granulomatosis with polyangiitis, which frequently demonstrates secondary orbital involvement from the nearby ethmoid or maxillary sinuses [9].

Initially our patient was treated with intravitreal bevacizumab injection OS to reduce the sub-retinal fluid associated with inflammatory CNVM. He required additional injections every 2–3 months throughout the course of treatment described in this case report, and he continues to be examined regularly by his retina specialist. The addition of orally administered prednisone to his treatment regimen after rule-out of infectious etiologies was crucial in managing the inflammatory component of his condition. His inflammatory CNVM showed signs of regression on clinical examination following immunosuppressive therapy with tapered steroids, and the frequency and quantity of new sub-retinal fluid decreased over time. With regard to his left upper eyelid blepharitis, his poor response to antibiotic ointment and warm compresses is consistent with IgG4-ROD, as well, and he experienced resolution of blepharitis after completion of an orally administered prednisone course. For patients with recurrent or refractory IgG4-RD, B cell depletion with rituximab appears to be a useful approach [3, 7, 8], and our case report is no exception. Rituximab infusions were initiated shortly after tonsillectomy in order to lower his elevated levels of circulating IgG4, and recurrence of disease was not observed.

This is the first reported case of inflammatory CNVM associated with IgG4-RD, a relatively new addition to the diagnostic lexicon that was perhaps previously misdiagnosed as IOI or some other variant of inflammatory orbital disease prior to quantitative IgG4 serum measurements and histopathological biopsy staining for IgG4. Despite the unusual presentation of IgG4-ROD as inflammatory CNVM with sub-retinal fluid and later with blepharitis, the course of disease and response to immunosuppressive treatment with steroids (and anti-VEGF therapy) described in this case report shows consistency with more common manifestations of ophthalmic IgG4-RD. Early detection of this disease is important to avoid organ damage and potential complications, so clinicians should maintain an index of suspicion for this condition when new inflammatory CNVM is observed in an otherwise atypical setting.

## Data Availability

The datasets used and/or analyzed during the current study are available from the corresponding author on reasonable request.

## Abbreviations

CNVM: Choroidal neovascular membrane
VEGF: Vascular endothelial growth factor
IgG4: Immunoglobulin G4
IgG4-RD: Immunoglobulin G4-related disease
OD: Right eye
OS: Left eye
OCT: Optical coherence tomography
ICG: Indocyanine green
IgG4-ROD: Immunoglobulin G4-related ophthalmic disease
IOI: Idiopathic orbital inflammation
RPE: Retinal pigment epithelium
